# QD laser eyewear as a visual field aid in a visual field defect model

**DOI:** 10.1038/s41598-018-37744-8

**Published:** 2019-01-30

**Authors:** Chigusa Iyama, Yuta Shigeno, Eri Hirano, Mamoru Kamoshita, Norihiro Nagai, Misa Suzuki, Sakiko Minami, Toshihide Kurihara, Hideki Sonobe, Kazuhiro Watanabe, Hajime Shinoda, Kazuo Tsubota, Yoko Ozawa

**Affiliations:** 1 Department of Ophthalmology, Keio University School of Medicine, Tokyo, Japan; 2 Laboratory of Retinal Cell Biology, Department of Ophthalmology, Keio University School of Medicine, Tokyo, Japan

## Abstract

Visual field defects interfere with free actions and influence the quality of life of patients with retinitis pigmentosa; the prevalence of this disease is increasing in aging societies. Patients with progressive disease may require visual aids; however, no such devices are currently available. We utilized a retinal projection eyewear system, QD laser eyewear, which includes a projector inside the spectacle frame, to draw the image taken by a connected portable camera with a wide field lens. The images are projected onto the retina using a Maxwellian view optical system, which is not influenced by refractive error or the amount of incident light. Goldmann perimetry and figure recognition tests with the QD laser eyewear showed increased visual field areas and angles, and shortened the time for recognition of the number of figures in a sheet, in a limited visual field model that we developed by using a pin-hole system to simulate the tunnel vision of retinitis pigmentosa in 19 healthy adults. The device supported the quality of vision. Additionally, the visual field defect model used in healthy adults was useful for validating the device in the development stage of the study, to clarify both advantages and future goals for improving the device.

## Introduction

Visual field defects caused by neurodegeneration remain an unresolved issue, despite recent advances in medical science. Such defects influence individuals’ quality of life (QOL) by interfering with free actions^1^. Retinitis pigmentosa is the leading cause of visual function impairment; the prevalence is approximately one in 3000−5000 individuals^2^, and is common as a hereditary disease. Retinitis pigmentosa is the third cause of blindness in Japan^3^, while glaucoma is the first, diabetic retinopathy is the second, and age-related macular degeneration (AMD) is the fourth cause^3^. Patients suffering from these diseases have difficulties in walking, driving, and reading^4–7^. These conditions pose a burden on society, given the increase in the numbers of such patients in aging societies. As yet, no curative treatments are available that can recover visual function, and therefore it would be helpful to provide supportive systems that can improve visual ability, including improving the visual field, to improve the QOL of these patients.

There have been several previous clinical studies of such visual aids^8–12^; however, these devices have not been introduced into daily use. A randomized study has investigated the use of prism glasses that project the image onto the retina outside of the degenerated lesion^13^, but found no significant effects. Other studies have used eyeglasses consisting of a see-through display and a camera that brightened the obtained images^14^, or a head-mounted depth camera and displays to brighten and present information about the distance of obstacles to the wearer^15^. However, many of these devices have not gained widespread use.

Here, we utilized a retinal projection eyewear system that contains a small projector inside a spectacle frame, to draw the image taken by a connected portable camera. More specifically, the image was projected onto the retina using the QD laser scanning system developed by QD Laser, Inc. (Kawasaki, Japan). In contrast to the typical displays in which optical systems are generated for emmetropic eyes, the greatest advantage of QD laser eyewear is that it employs a Maxwellian view optical system. In this system, a real image of a light source is focused on the pupil of the eye, and the image reaches the retina without defocusing, irrespective of the axial length or refractive indexes of the ocular parts; there is thus no need to correct refractive error. In addition, pupil size does not influence the amount of light entering the eye. The Maxwellian view optical system is often used to present stimuli for eye examinations, e.g., to analyse the optical density of the lens^16^, macular pigment^17,18^, colour appearance^19^, cone function^20^, photo stress-recovery related to coloured intraocular lenses^21^, light adaptation^22^, and localization of photophobia^23^. Moreover, the resolution of the projector of QD laser eyewear is as high as 1000 × 600, and that of the camera is 2 million pixels.

The projector is equipped with a microelectromechanical system through which a laser scans the retina, without diffusion; thus, it reflects colour clearly. The eyewear is categorized among Class I laser products, which are considered to be safe under reasonably foreseeable conditions of operation, including the use of optical instruments for intrabeam viewing, based on the international standard for laser safety (IEC 60825-1) established in 2014. In the laser, low-power red, green, and blue laser light oscillate; the blue light wavelength is at 1/40, and that of red and green is at 1/400 the power of the maximum levels permitted by the Japanese Industrial Standard and International Electrotechnical Commission.

In this study, we developed a model of patients with a limited visual field by using a pin-hole system in healthy adults, which may mimic late-stage retinitis pigmentosa, and analysed the effects of using the QD laser eyewear carrying a wide-angle lens on the camera. The results of this study may facilitate the development of a new device for individuals who have a visual field defect, such as patients with retinitis pigmentosa. We also discuss the value of our newly developed visual field defect model for optimizing the new device.

## Results

**Figure 1:**
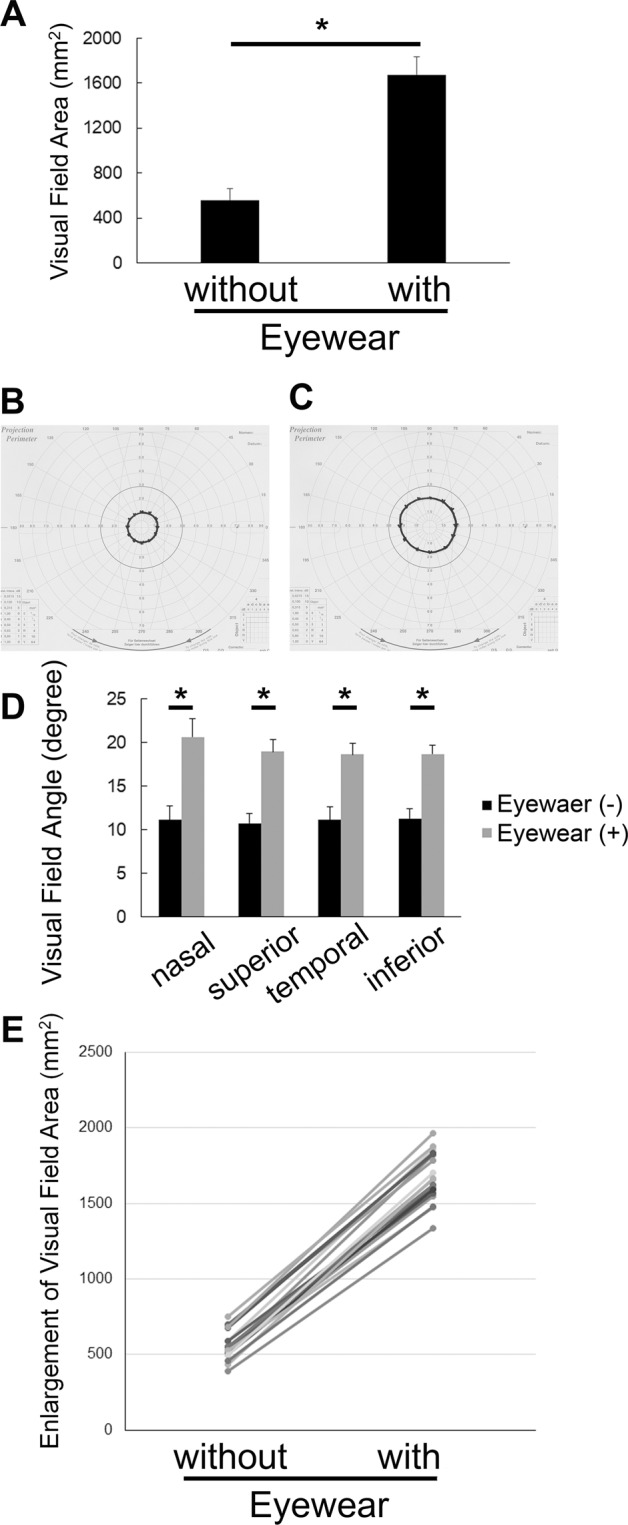
Enlargement of the visual field by the QD laser eyewear, as measured by the Goldmann perimetry test. (**A−E**) The mean visual field area (**A−C**) and angles (**D**), limited by using a pin-hole attachment in healthy volunteers, was enlarged by the QD laser eyewear. Representative results of Goldmann perimetry results without (**B**) and with (**C**) use of the QD laser eyewear, in addition to a pin-hole attachment, in the same individual. All individuals showed an enlargement of the area (**E**). The lines in the graph show the data without and with QD laser eyewear of the individuals. (**E**) *P < 0.001.

The examinations were performed in only the right eyes of 19 participants, whose left eyes were masked with a black obstacle. Although the system is not influenced by refractive error, we asked participants to wear contact lenses if needed, to ensure best-corrected visual acuity, and to equalize conditions between individuals as far as possible. The mean visual field area of the participants, wearing a pin-hole attachment to limit the visual field artificially, was recorded using a stimulus intensity and size of V/4 in Goldmann perimetry; this area measured in the output result sheet was 561.0 ± 98.4 mm^2^ in our healthy volunteers (Fig. 1A,B, Table 1). However, utilizing the QD laser eyewear significantly increased the mean visual field area by 303.3 ± 35.4%, to 1674.5 ± 163.3 mm^2^ (Fig. 1A,C). The mean angle in each direction of the visual field was increased (Fig. 1D, Table 1). The angles of the individuals with a pin-hole attachment and without the QD laser eyewear were not greater than 15 degrees in every direction, and was tunnel vision; however, that with the pin-hole attachment as well as QD laser eyewear was greater than 15 degrees (Supplementary Table 1), resolving the issue of tunnel vision, and making it clinically relevant. Thus, enlargement of the visual field was obtained by using QD laser eyewear, in all individuals (Fig. 1E).

**Table 1:** Improvements of visual ability by QD laser eyewear.

	with a pin-hole		P value
	without QD laser eyewear	with QD laser eyewear	
***Goldmann perimetry***			
visual field area (mm^2^)	561.05 ± 98.40	1674.47 ± 163.33	< 0.001*
**visual field angle (degree)**			
nasal	11.11 ± 1.59	20.63 ± 2.09	<0.001*
superior	10.74 ± 1.15	18.95 ± 1.35	<0.001*
temporal	11.11 ± 1.52	18.63 ± 1.26	<0.001*
inferior	11.32 ± 1.11	18.68 ± 1.06	<0.001*
***Figure recognition tests*** ***Time to recognize the number of each target figure in the sheet (sec)***			
●	3.62 ± 0.52	2.84 ± 0.39	<0.001*
▲	3.94 ± 0.85	3.26 ± 0.63	<0.001*
**++**	3.64 ± 0.64	3.03 ± 0.51	<0.001*
★	3.92 ± 0.63	3.40 ± 0.66	<0.001*

Data are mean ± SD. Mann-Whitney test was applied to assess visual field angles in Goldmann perimetry tests with or without QD laser eyewear, and Student’s *t*-test was applied for the other analyses. *P<0.001.

**Figure 2:**
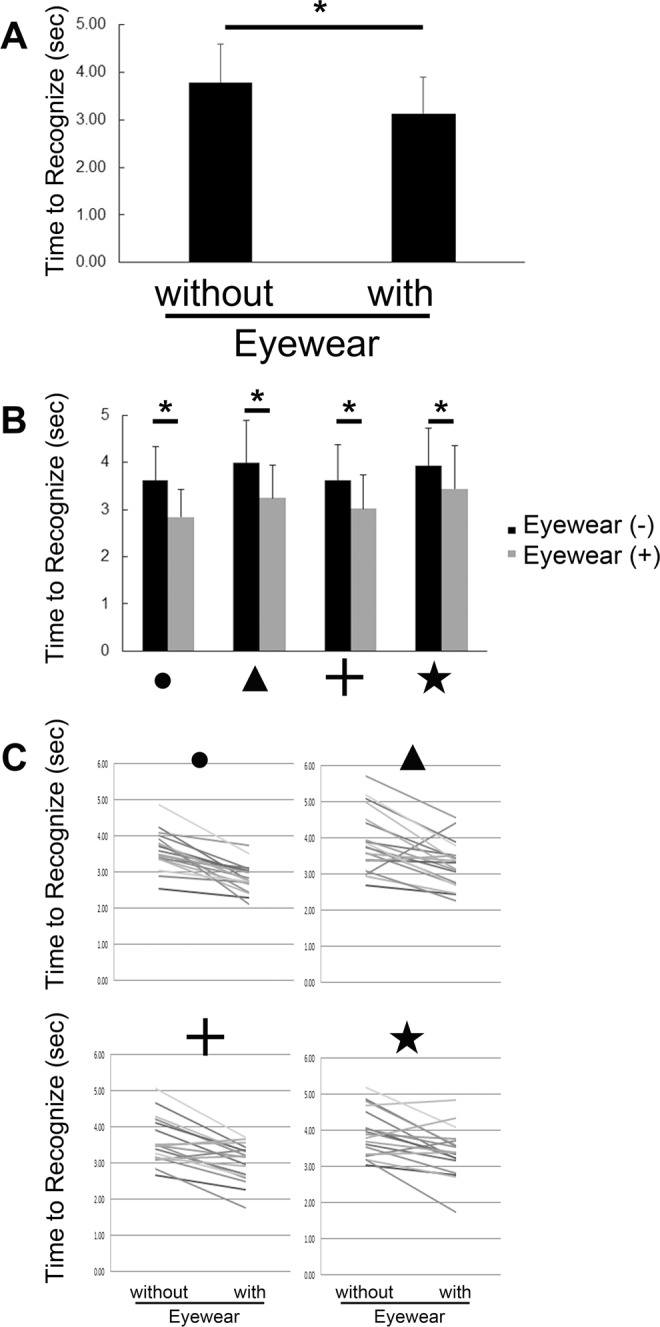
Rapid answers in the figure recognition test obtained when using the QD laser eyewear. (**A**) The average duration for recognition and counting of the number of each figure on a sheet was shortened when using the QD laser eyewear. (**B**) The duration was significantly shortened for each figure. (**C**) The duration was significantly shortened in most of the each individual; the lines in the graph show the data without and with QD laser eyewear of the individuals. ● of, +, and ★ are the target figures. *P < 0.001.

Then, we performed figure recognition tests with or without QD laser eyewear, in addition to the pin-hole attachment and best-correction contact lenses. Participants were asked to count the number of the respective target figures on a sheet on the wall. There were three types of sheets, each of which had different alignments of the four types of target figures, and all the participants reported the number of target figures counted on each sheet. The number of figures on the three sheets randomly shown were first reported without QD laser eyewear, and subsequently with QD laser eyewear; the order in which the three sheets were shown in both examinations were always random. The overall average time required to recognize and count the numbers of four target figures was 3.79 ± 0.8 s with the pin-hole attachment, but without the QD laser eyewear. In contrast, the time required was significantly shorter, at 3.13 ± 0.8 s, when the QD laser eyewear was also worn (Fig. 2A, Table 1); the reduction in average time was obtained in 18 out of 19 individuals. The reduction in time was significant for all types of figures (Fig. 2B, Table 1). The number of individuals who obtained the reduction in time for each figure with QD laser eyewear was as follows; ●, 19; ▲, 17; +, 15; ★, 15 out of 19 participants (Fig. 2C, Supplementary Table 2). If the answers were incorrect, the data of the time to count the same figure in the same sheet, both with and without QD laser eyewear, were excluded. The correct answer rate for each figure when using the QD laser eyewear was as follows: ●, 95%; ▲, 71%; +, 93%; ★, 91%, and without the QD laser eyewear it was: ●, 100%; ▲, 86%; +, 100%; ★, 96%.

There were no adverse events reported by the participants.

## Discussion

We here report that using QD laser eyewear (carrying a wide-angle lens in the camera) resulted in a significant improvement in the visual field and in the time required to recognize the number of figures arranged on a sheet, in a model of a visual field defect generated by using a pin-hole attachment in healthy volunteers.

The participants in this study were all healthy adults; however, the use of a pin-hole attachment behind the eyewear enabled us to generate a model of a visual field defect. It is not practically easy to collect patients with a visual field defect who are at a similar pathological stage. A previous study that evaluated a new visual aid device, which used a see-through display, included only eight patients^14^, while a study investigating the use of residual vision glasses that consisted of a pair of organic light emitting diode (OLED) panels and an infrared depth camera, mounted within an adjustable headset, included only 11 patients^15^. Patients in such studies may have various degrees of visual field defects, as well as variation in other visual functions, which may have confounding effects on the results; nevertheless, knowing the experience of each of the patients may also be of value to improve the device. In contrast, the model that we generated in the current study was based on healthy adults with relatively homogenous visual function. Therefore, the data variation was small and allowed us to define the advantages and future goals for improving the device clearly, as described below. This model can contribute to future studies seeking to validate visual aid devices, although the final trials should involve actual patients.

Using the QD laser eyewear carrying a wide-angle lens, the visual field area was increased in all subjects in the current study. Projection of the image through the wide-angle lens to the limited area of the display was made possible by the high resolution of the device (1000 × 600 pixels), which was approximately 30 times greater than that used in a previous study (160 × 128 pixels)^15^. We are currently working on further improvement of resolution for use of the device by patients who have reduced retinal sensitivity.

The advantages of this device were also demonstrated by the figure recognition test. Participants required less time to count the number of figures arranged on a sheet, most likely because the whole sheet was observable at a glance, without needing to move the camera to search for the targets. However, the correct answer rates varied according to the shape of the target; ▲tended to be misrecognized. In addition, four participants required more time to recognize the + and ★ targets with the device. Further improvement in the resolution and clarity of the image may be required to allow precise distinction of targets. Patients might have less sensitivity than healthy adults even in the responsive areas of the retina that comprise the visual field. The phenomena noted above may have been related to the resolution of the camera, rather than the projection system. A more recent model of the eyewear established by QD Laser, Inc. for general rather than medical use can mount a 4 K camera, which can project clearer images, although it is not currently portable (personal communication). Thus, the above problem is most likely to be resolved in the near future by mounting a higher-grade camera in the medical use-QD laser eyewear.

We are currently making the personal computer required for use with this device portable, for more convenient use in daily life, e.g., when a patient visits a place for the first time. We considered that this device may be useful for reading price tags or documents outside of the daily environment, rather than for reading a book. Reading a book at home could be more efficiently supported by a text-to-sound or -voice application on a personal computer. Visual function impairment can cause vision-related impairment of QOL^24^, and obtaining a visual field with a view greater than 20 degrees may be useful for walking^25^. The current device that improved the visual field to almost 20 degrees would, at least in part, have a clinically relevant effect.

Our study also demonstrated the safety of the device: there were no adverse events, such as eye troubles or nausea, reported in the current study. However, because in real-world clinical practice, the device may be used throughout the day, and not only during an eye test as in the current study, long-term observation is required.

Recently, cell biology has led to advances in regenerative medicine. Transplantation of retinal cells may become a therapeutic option in future. However, the use of the proposed device to maximize the residual ability would provide non-invasive beneficial effects for greater numbers of patients. The targeted patients in the current study were those with retinitis pigmentosa; however, future studies might expand the application of the device to AMD patients who have a limited, but less-affected, function-retaining area at the central retina.

The study had the following limitations. The number of participants was relatively low, in contrast to a previous randomized controlled trial of prism spectacles for AMD^13^. In our study, participants had healthy retinas, although they had tunnel vision due to wearing the pin-hole attachment. The final goal is to apply the device in patients, such as those with retinitis pigmentosa, who may also have impaired visual sensitivity. The resolution of the device may thus also be important. However, it may be easier to test the effects and identify issues in the device by using a healthy adult-derived disease model. Another limitation is that the tests were performed during the first use of the device by the participants, while learning and training may be necessary to ensure smooth use of the device. However, our participants clearly obtained beneficial effects, even though they had no previous learning experience with this device. The data of target recognition through the pin-hole was obtained first without and then with the device; thus, we cannot exclude a possibility that the reduced time with the device involved a learning effect, although trial examinations were performed before data sampling in individuals, and the data obtained for the three examinations under each condition (without or with the device) did not vary within individuals, suggesting that the learning effect was small, if any. The current study did not include orientation and mobility training, which is an important measure from the patient’s point of view.

In summary, QD laser eyewear enlarged the visual field three-fold and shortened the time required for recognizing targets in a visual field defect model that was artificially generated in healthy adults. The model that we established in the current study was useful for validation of the device in the development stage, and allowed identification of the advantages and future goals for improvement of the device. This study forms the basis for development of a new low-vision aid device that can support the quality of vision, thereby, at least in part, contributing to improved QOL of patients who have a limited visual field, such as those with retinitis pigmentosa, in future.

## Methods

### Ethics

This study adhered to the tenets of the Declaration of Helsinki, and was approved by the Ethics Committee of Keio University School of Medicine (approval number: 20160011), and registered as UMIN 000017846 (09/06/2015). Written informed consent was obtained from all subjects.

### Study participants

Nineteen healthy adult volunteers (8 men, 42%) ranging from 22 to 31 years of age (average 26.4 ± 2.7 years) were analysed at the Department of Ophthalmology, Keio University Hospital, from June to December 2016. None of the participants had ocular diseases, except for refractive error that was corrected by contact lenses, and not by glasses, to ensure that they could wear the QD laser eyewear easily. A sample size estimation was not performed, and we simply used the number of research participants who applied in response to our recruitment requests. The best-corrected visual acuity (BCVA) of all the participants were 1.2 in decimal (−0.079 in logMAR); this was a point of difference from real patients with retinitis pigmentosa who would have less sensitivity in the remaining area of the retina, and was a limitation of this study. At the end of the study, they were asked whether they had experienced any adverse events.

### QD laser eyewear and attachments

**Figure 3:**
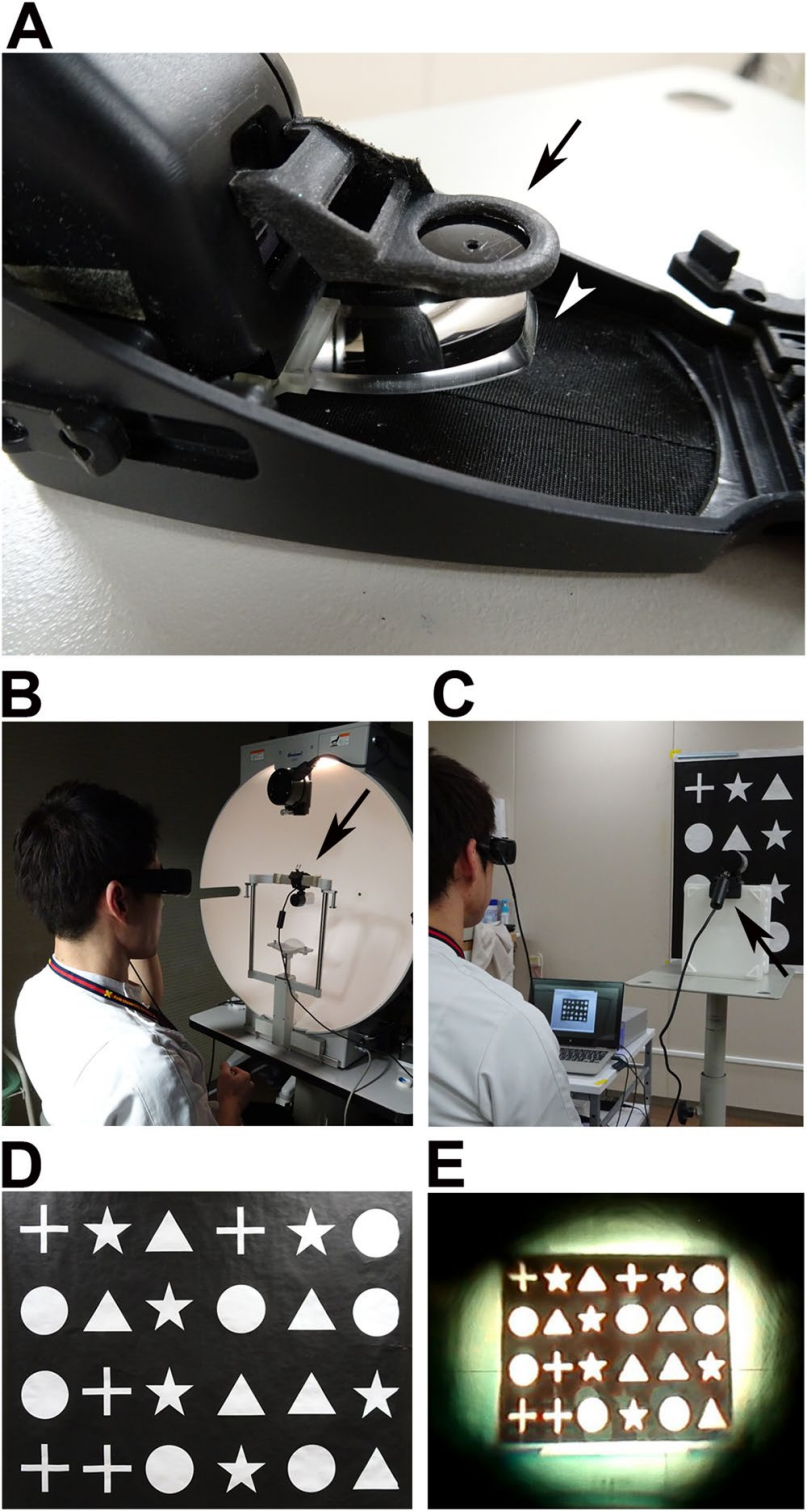
The laser eyewear and examinations. (**A**) The laser eyewear with a pin-hole attachment. Black arrow shows the pin-hole, and the white arrowhead shows the display of the laser eyewear behind the black obstacle for the right eye. (**B**) The camera, with a wide-angle lens, was set above the face table during the Goldmann perimetry test. The arrow shows the camera with a wide-angle lens. (**C**) The camera with the wide-angle lens (arrow) was set during the figure recognition test. (**D**) One of the figure sheets for the figure recognition test. (**E**) The image shown on the display during the figure recognition test. The whole sheet was observable at a glance using the QD laser eyewear system with a wide field lens.

All participants wore the eyewear with a pin-hole attachment to limit their visual field artificially during all the examinations, with and without the QD laser eyewear, a retinal projection laser eyewear system (QD Laser, Inc., Kawasaki, Japan) (Fig. 3A). The tests were performed only in their right eyes, instead of in the dominant eyes of participants in whom both eyes were healthy, with the other eye masked by a black obstacle. The device carries a small projector inside the frame for the right eye, to draw the image taken by a connected portable camera, and is a type of head-mounted display. In this device, retinal scan technology, named “VISIRIUM® Technology” (QD Laser), is applied. In the current study, a 2.6 (vertically) × 2.6 (horizontally) wide-angle lens (providing a 6.76 times wider area) was attached to the camera to obtain a wider visual field (Fig. 3B,C). The system uses a Maxwellian view optical system, and thus the participants theoretically had no need to use refractive correction; however, they were asked to use contact lenses for best-correction of visual acuity. Informed consent for an online open-access publication of the image was obtained from the subject in Fig. 3.

### Goldmann perimetry tests

The participants underwent Goldman perimetry tests using a stimulus intensity and size of V/4, with and without laser eyewear, in addition to the pin-hole. The examinations were performed by orthoptists (YS and EH). During the tests, the camera of the laser eyewear, instead of the participants’ face, was set above the face table (Fig. 3B). Visual field was assessed by the area recorded in the standard output result sheet measured using ImageJ software (https://imagej.nih.gov/ij/index.html), as described in a previous report^26^, and the angles of nasal (0°), superior (90°), temporal (180°), and inferior (270°) areas. Under this calibration, an area of 556 pixels was equivalent to 1 mm^2^ on the visual field recording paper.

### Figure recognition tests

The participants were asked to count the number of each figure in the sheets posted on the wall, with or without wearing the laser eyewear in addition to the pin-hole attachment (Fig. 3C). Each sheet involved four types of randomly aligned figures (Fig. 3D,E), and three patterns of sheets with different alignments were prepared to determine the time required to answer. The results were averaged and compared between tests with and without the laser eyewear. During the tests with the laser eyewear, the camera of the laser eyewear was set at 2.3-m distance from the wall and at a height of 1.3 m, to adjust to the position of the eyes of the participants (Fig. 3C).

### Statistical analyses

Data are expressed as means ± standard deviations (SDs). Commercially available software (IBM SPSS Statistics, version 24.0, IBM Japan, Tokyo, Japan) was used for all statistical analyses. Mann-Whitney test was applied to assess visual field angles in Goldmann perimetry tests with or without QD laser eyewear, and Student’s *t*-test was applied for the other analyses. P-values of < 0.05 were considered statistically significant.

## Supplementary information

Supplementary Tables 1 and 2

## Data Availability

The protocol and the datasets generated during and/or analysed during the current study are available from the corresponding author on reasonable request.
