# Supplementary material for: QD laser eyewear as a visual field aid in a visual field defect model
Source: Sci Rep. 2019 Jan 30;9:1010. doi: 10.1038/s41598-018-37744-8 (PMC6353865; doi:10.1038/s41598-018-37744-8)
Supplement: Supplementary file 1 — Supplementary Tables 1 and 2 [file 41598_2018_37744_MOESM1_ESM.pdf]

QD laser eyewear as a visual field aid in a visual field defect model

Chigusa Iyama 1a¶, Yuta Shigeno 1a¶, Eri Hirano 1a¶, Mamoru Kamoshita 1a,1b, Norihiro Nagai 1a,1b, Misa Suzuki 1a,1b, Sakiko Minami 1b, Toshihide Kurihara 1b, Hideki Sonobe 1b, Kazuhiro Watanabe 1a, Hajime Shinoda 1a, Kazuo Tsubota 1a, Yoko Ozawa 1a,1b*

¶ Equally contributed first authors

1a Department of Ophthalmology, 1b Laboratory of Retinal Cell Biology, Department of Ophthalmology, Keio University, School of Medicine

Financial support: QD Laser, Inc., Kawasaki, Japan

Running head: QD eyewear as a visual field aid

***Corresponding author:**

Yoko Ozawa, M.D., Ph.D.

Laboratory of Retinal Cell Biology

Department of Ophthalmology

Keio University School of Medicine

35 Shinanomachi, Shinjuku-ku, Tokyo 160-8582, Japan

Phone: +81-3-5363-3869, Fax: +81-3-5363-3869

E-mail: ozawa@a5.keio.jp

Supplementary Table 1. Visual field angles with or without QD laser eyewear in addition to a pin-hole in individuals (degree)

	nasal		superior		temporal		inferior	
participants	without QD	with QD	without QD	with QD	without QD	with QD	without QD	with QD
1	10	23	10	20	10	20	11	18
2	9	18	11	18	12	20	12	18
3	10	22	10	17	11	19	10	20
4	11	22	11	20	11	19	11	20
5	10	22	10	17	10	17	11	18
6	12	22	11	20	14	20	13	20
7	11	22	12	21	15	20	12	19
8	11	18	11	18	11	18	12	20
9	12	19	12	20	11	21	11	17
10	12	21	12	20	10	17	11	17
11	11	20	10	18	10	17	11	19
12	15	23	10	20	12	18	14	19
13	14	20	11	20	13	20	11	20
14	13	24	14	20	11	19	13	18
15	11	18	10	19	11	18	11	18
16	10	22	10	20	10	17	11	18
17	9	17	9	18	9	18	10	18
18	10	21	10	17	10	18	10	20
19	10	18	10	17	10	18	10	18

QD, QD laser eyewear.

Supplementary Table 2. Mean time to recognize the number of each target figure in the sheet in individuals (sec)

participants	●		▲		+		★	
	without QD	with QD	without QD	with QD	without QD	with QD	without QD	with QD
1	3.37	3.12	3.74	3.08	3.92	2.97	3.63	3.18
2	3.60	3.04	3.89	3.51	3.40	2.68	3.96	3.35
3	4.01	3.10	4.43	3.45	4.22	3.22	4.51	3.21
4	3.46	3.00	3.94	3.13	3.48	3.16	3.89	3.77
5	2.54	2.29	2.69	2.43	2.66	2.28	3.04	2.76
6	4.24	2.76	5.11	3.90	4.66	3.44	4.86	3.56
7	2.88	2.72	2.98	4.41	3.10	3.36	3.29	3.71
8	3.80	2.43	3.58	2.72	3.52	2.60	3.55	2.82
9	4.09	3.74	5.72	4.58	4.21	3.23	4.83	3.60
10	3.78	2.76	5.01	3.37	4.29	3.32	4.70	4.84
11	3.72	2.98	3.41	3.32	4.12	3.35	4.06	3.24
12	3.42	2.85	3.76	2.76	3.16	2.49	4.03	3.55
13	3.50	2.99	3.36	3.51	3.11	3.20	3.69	3.39
14	3.38	2.66	3.58	3.37	3.51	3.56	3.33	3.40
15	3.36	2.41	2.95	2.46	3.14	2.92	3.19	2.73
16	4.87	3.51	5.19	3.79	5.08	3.73	5.19	4.09
17	3.92	2.12	3.09	2.27	2.85	1.77	3.20	1.73
18	3.78	2.77	4.53	3.14	3.46	3.67	3.80	4.35
19	3.05	2.74	3.86	2.69	3.27	2.64	3.81	3.37

QD, QD laser eyewear.
